# Robust optimization through neuroevolution

**DOI:** 10.1371/journal.pone.0213193

**Published:** 2019-03-01

**Authors:** Paolo Pagliuca, Stefano Nolfi

**Affiliations:** Institute of Cognitive Sciences and Technologies—National Research Council (CNR), Via S. Martino della Battaglia, Roma, Italia; Sichuan University, CHINA

## Abstract

We propose a method for evolving neural network controllers robust with respect to variations of the environmental conditions (i.e. that can operate effectively in new conditions immediately, without the need to adapt to variations). The method specifies how the fitness of candidate solutions can be evaluated, how the environmental conditions should vary during the course of the evolutionary process, which algorithm can be used, and how the best solution can be identified. The obtained results show how the method proposed is effective and computational tractable. It allows to improve performance on an extended version of the double-pole balancing problem, outperform the best available human-designed controllers on a car racing problem, and generate effective solutions for a swarm robotic problem. The comparison of different algorithms indicates that the CMA-ES and xNES methods, that operate by optimizing a distribution of parameters, represent the best options for the evolution of robust neural network controllers.

## 1. Introduction

Many real-world optimization problems require the development of systems capable of operating in environmental conditions that change stochastically or dynamically over time [1, 2, 3]. The physical world is full of uncertainties and almost no parameter, dimension, or property tends to remain exactly constant. Machines may break down or wear out slowly, raw material is of changing quality, the physical conditions of the environment change continuously as a result of meteorological phenomena or the activities of other systems, new jobs might need to be integrated with activities already scheduled.

To operate effectively in variable environmental conditions systems should be able to adapt dynamically to environmental variations or should be robust, i.e. they should be able to operate as satisfactory as possible in varied environmental conditions immediately, without the need to further adapt. In certain conditions, e.g. when the environment changes too quickly, when the environment cannot be monitored closely enough, or when the cost associated to the time required to adapt to the new environmental conditions is too high, robustness represents the only possible way to handle the effect of environmental variations [3].

In this paper we investigate the use of artificial evolution for robust design optimization [4], namely the synthesis of solutions capable of operating satisfactorily in varied environmental conditions immediately. This is investigated in the context of neuro-controlled agents evolved in varying environmental conditions. We decided to use agents situated in an external environment since it permits to study the effect of environmental and agent/environmental variations. For examples of other possible application domains see [5–7]. Moreover, we decided to use neuro-controlled agents to exploit the ability of neural networks to regulate their output on the basis of their input and generalize, i.e. respond to new inputs with outputs similar to those produced for similar inputs. Regulating the output on the basis of the perceived environment allows to display multiple behaviors and select the behavior that is appropriate to the current environmental circumstances [8]. The tendency to respond to new inputs with outputs similar to those produced for similar inputs increases the chance to master appropriately new environmental conditions without the need to adapt to them.

After reviewing the previous works carried out in this area, we introduce a method that can be used to evolve robust solutions. He method specifies: how the fitness of candidate solutions can be evaluated, how the variation of the environmental conditions during the course of the evolutionary process can be controlled in order to improve the quality of the evolved solutions, how the best solution of a run can be identified, and which are the characteristics that the evolutionary algorithm should have to operate effectively in varying environmental conditions.

The method is validated on three qualitatively different problems: (1) an extended version of the double-pole balancing problem, (2) a car-racing problem, and (3) a swarm robotic problem.

Results are collected by using a standard (*μ* + *μ*) evolutionary strategy [9, 10] and four state-of-the-art evolutionary algorithms: (1) the covariance matrix adaptation evolution strategy (CMA-ES, see [11]), (2) the exponential natural evolution strategy (xNES, see [12]), (3) the separable natural evolution strategy (sNES, see [12]), and (4) the neuroevolution of augmenting topologies (NEAT, see [13]).

The obtained results show how solutions displaying a high level of robustness can be obtained by evaluating candidate solutions in a relatively small number of different environmental conditions. Moreover, the results show that the CMA-ES, xNES and sNES algorithms, that operate by optimizing distribution of parameters, outperform alternative algorithms operate by optimizing specific combination of parameters.

## 2. Related research

**Fig 1:**
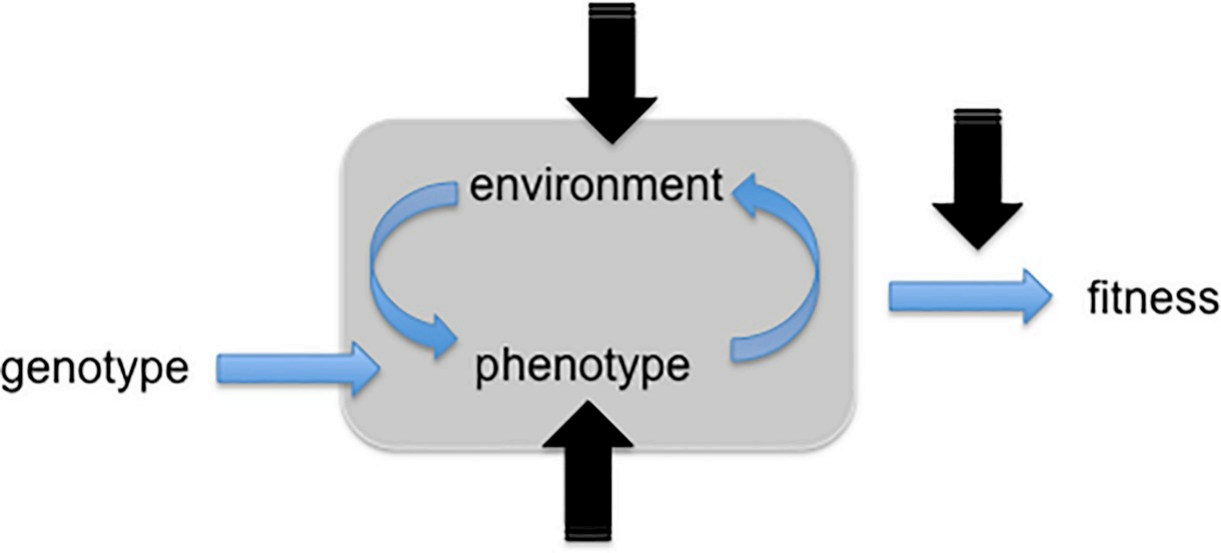
Schematization of the components of an evolutionary systems subject to variations. The genotype determines the characteristics of the phenotype (solution). The continuous interactions between the phenotype and the environment produce a behavior (represented by the grey rectangular area). The fitness indicates the efficacy of the behavior. As indicated by the black arrows, variations might affect the phenotype (i.e. the internal environment), and/or the environment (i.e. the external environment or the relation between the agent and the environment), and/or the fitness evaluation. Adapted from Figure 8.3 included in [3].

Uncertainties in evolutionary systems embedded in an external environment can occur as a consequence of variations affecting: (i) the internal environment of the candidate solution, and/or (ii) the external environment, and/or (iii) the evaluation of the objective function (see Fig 1) [3, 4]. “The goal of robust optimization therefore is twofold: (1) to find optimal solution despite uncertainties and noise in the optimization model, and (2) to find optimal solutions that are robust with respect to uncertainties and noise, and therewith useful in practice.” [14], pp. 213.

The most general approach to evolve robust systems consists of evaluating candidate solutions multiple times in varying environmental conditions, i.e. carrying out multiple evaluation episodes characterized by different environmental conditions. Different types of robustness can be promoted through the usage of fitness functions rewarding for: (i) the maximization of the expected fitness, i.e. the average fitness obtained in all possible environmental conditions [3], (ii) the maximization of the worst-case fitness [15], (iii) the minimization of the variations of fitness in varying conditions [3], (iv) the maximum of the amount of tolerable disturbances [16], (v) the maximization of the ratio between the standard deviation of the fitness and the amount of environmental variation [17]. When the fitness function returns a single value, a standard evolutionary algorithm is used [8, 18, 19]. Instead, when the fitness function returns multiple values, e.g. the expected fitness and the fitness variance, the usage of a multi-objective evolutionary algorithm can be beneficial [20, 21–23]. In our experiments we will consider the optimization of the expected fitness only.

Overall, the evolution of robust solutions requires the determination of the following four aspects: (1) how the fitness is estimated, (2) how the environmental conditions are varied (at least in the case in which the experimenter has the possibility to manipulate them), (3) how the best solution of a run is identified, and (4) which evolutionary algorithm is used. In the rest of this section we discuss related research for each of these four aspects. The evidences collected in this body of research and in the experiments that we report in this paper are at the basis of the formulation of the original method that we propose, which is described in section 3.

For what concerns the first aspect, the most obvious option consists of evaluating candidate solutions in all possible environmental conditions. An exhaustive evaluation of this type, however, is possible only when the number of environmental parameters that can vary and the number of states that they can assume are small. In the other cases, fitness should be estimated by evaluating candidate solutions on a subset of all the possible environmental conditions. The higher the number of evaluation episodes carried out under different environmental conditions, the better the precision of the estimation. On the other hand, the higher the number of evaluation episodes, the higher the computational cost. Therefore, one should identify a good balance between accuracy and cost. The problem of measuring fitness in variable environmental conditions should not be confused with the problem of estimating fitness in the presence of measuring errors. Indeed, in the latter case, each candidate solution has a single fitness level affected by measuring errors. Instead, in the former case each candidate solution has N potentially different fitness levels in N different corresponding environmental conditions. Consequently, in case of variable environmental conditions, we cannot directly apply the techniques that can be used to minimize the number of evaluation episodes while ensuring a sufficient precision of the fitness estimation in the case of noisy fitness functions. Examples of such techniques include: (i) the usage of statistical tests to identify the minimal number of evaluations that can be used to differentiate with a sufficient level of precision the relative fitness of alternative candidate solutions [24, 25, 26], (ii) the usage of a large population size that permits to average implicitly the outcome of the evaluations carried out on different but similar candidate solutions [1, 27], (iii) the utilization of a non-zero threshold for accepting offspring [28], and (iv) the re-usage of the outcome of the evaluation of similar candidate solutions [29, 30]. Some researchers succeed in evolving solutions robust with respect to environmental variations by using a single evaluation episode conduced in randomly selected environmental conditions. For example, [31] manage to successfully evolve neural controllers for Gym-v1 continuous control benchmarks, which are robust with respect to small perturbations of their initial posture. As pointed out by [32], however, this only permits to tolerate a rather limited amount of variation. Solutions robust to wider environmental variations can only be obtained by exposing candidate solutions to stronger environmental variations and by conducting multiple evaluation episodes.

Regarding the second aspect, namely the selection of the conditions in which candidate solutions are evaluated, one should determine how the conditions experienced by each candidate solution are chosen and whether the conditions vary among the individuals of the same population and across generations. The simplest way to choose the conditions in which candidate solutions are evaluated consists of selecting them randomly with a given distribution (e.g. uniform or Gaussian). This can be achieved by setting the value of each aspect subject to variation randomly within a given variation range at the beginning of each evaluation episode. Alternatively, variance reduction techniques, such as Latin hypercube sampling (LHS), can be applied [28, 33]. The fitness of candidate solutions evaluated in easier and harder conditions tend to be over-estimated and under-estimated, respectively. These over and under-estimation effects create a noise in the fitness estimation unless the candidate solutions of each generation are evaluated on the same type of varying environmental conditions [28, 33]. Varying the environmental conditions across generations should be beneficial since it reduces the probability to retain candidate solutions performing well on the current environmental conditions but poorly in other environmental conditions. As shown by [34], the benefit might also depend on the frequency of variation of environmental conditions across generations and can be maximized at moderate frequencies (i.e. by varying the conditions every N generations rather than every generation).

The third aspect refers to the identification of the best solution of an evolutionary run. Since the performance depends on the environmental conditions, selecting the candidate solution that achieved the best fitness during the evolutionary process does not guarantee to select the best solution or at least one of the best solutions, i.e. the solution that would achieve the best average performance in all possible environmental conditions. Indeed, the solution with the best fitness during the course of the evolutionary process typically encountered favorable environmental conditions but performs much poorly in other conditions. This problem can be solved by post-evaluating evolved solutions in new environmental conditions and selecting the candidate solution displaying the best performance during post-evaluation. To limit the computational cost required by post-evaluation, one can restrict the post-evaluation to the candidate solutions of the last generation (see for example [10]) or to the fittest candidate solution of each generation.

Finally, the fourth aspect concerns the properties that the evolutionary algorithm should have to successfully synthesize robust solutions. Firstly, the selective pressure of the algorithm should be sufficiently strong to compensate the implicit reduction caused by the fact that the fitness represents only an estimation of performance. Secondly, the algorithm should re-evaluate the fitness of candidate solutions that remained unchanged from previous generations as a result of elitism or steady state selection. This is required to avoid the un-justified permanence over generations of candidate solutions with over-estimated fitness. Finally, certain types of evolutionary algorithms might result more suited for the evolution of robust solutions than others. In particular, methods that optimize the distribution of parameters (e.g. CMA-ES, xNES, and sNES; [11, 12]) can be more effective than techniques optimizing specific set of parameters (e.g. standard Evolutionary Strategies [35] or NEAT [13]). As pointed out by [36], methods optimizing a distribution of parameters tend to select solutions robust with respect to parameter variations. Although robustness to variations of the parameters and robustness to variations of the environmental conditions are qualitatively different, solutions that are robust to the former tend to display a certain robustness to the latter too [37]. Furthermore, the incremental estimation of the gradient across generations, in the presence of variable environmental conditions, implicitly produces the estimation of a gradient averaged over variable conditions. This might drive the optimization process toward areas of the search space containing robust solutions. Instead, a potential drawback is constituted by the fact that the need to estimate the gradient in varying environmental conditions might reduce the accuracy of the estimation.

The problem of evolving robust solutions in varying environments presents similarities with the problem of evolving solutions in environments where the environmental conditions are static but the fitness measure is noisy [1]. Indeed, both problems usually require the usage of multiple evaluation episodes. As pointed out above, however, the two problems also present differences. In particular, the perturbations affecting the fitness measure usually have zero mean, are symmetric, have a regular distribution, and have effects independent of the characteristics of the candidate solution. On the contrary, the effects of environmental variations on the fitness have non-zero mean, are asymmetric, have a skewed distribution, and strongly depend on the characteristics of the candidate solution [3].

Finally, the study of evolutionary robust optimization is related to the study of evolutionary dynamic optimization [1, 2, 38], namely the study of how an optimization algorithm can solve an optimization problem in a given period [t_begin_, t_end_] during which the underlying fitness landscape changes and the optimization algorithm reacts to such changes by generating new optimal solutions (adapted from definition 1 included in [38]). Indeed, in both cases the characteristics of the environment vary. Nevertheless, the objectives of robust and dynamic optimization are different. Robust optimization aims to develop solutions capable of operating as effectively as possible in new environmental conditions immediately, without further adaptation. Dynamic optimization seeks to develop solutions that are not necessarily effective after the variation of the environment, but can adapt to the new environmental conditions as readily as possible [39]. Consequently, also methodological issues differ. For example, mechanisms preserving population diversity can speed-up the evolution of agents capable of adapting to the new environmental conditions [2, 38], but they are not necessarily important for the evolution of robust agents. Moreover, the formalization of a method for measuring the speed with which agents adapt to the new environmental conditions is crucial for dynamic optimization [2, 38], but it is not relevant for the study of robust agents.

### 3. A method that enables the evolution of robust solutions

In this section we describe the method that we propose to evolve robust solutions, the rationale behind it, and the relation of the method with pre-existing related techniques.

Candidate solutions are evaluated multiple times in randomly varying environmental conditions. The variables subject to variations can include the initial relation between the agents and the environment, e.g. the initial position and orientation of the agents in the environment, and/or the characteristics of the environment, e.g. the position and orientation of certain environmental objects. Variations are introduced by generating an environmental variation matrix (EVM) with NEE columns and NP rows, where NEE corresponds to the number of evaluation episodes and NP to the number of environmental parameters subjected to variation. The values of the matrix are generated randomly with a uniform distribution within the range of variation of the environmental parameters ([P1min, P1max], …. [PNmin, PNmax]). At the beginning of each episode the environmental conditions are set on the basis of the corresponding row of the matrix. EVM is regenerated across generations at a certain frequency F, i.e. the environmental conditions vary across generations with a certain rate. The fitness of candidate solution is computed by averaging the fitness obtained during evaluation episodes.

**Fig 2:**
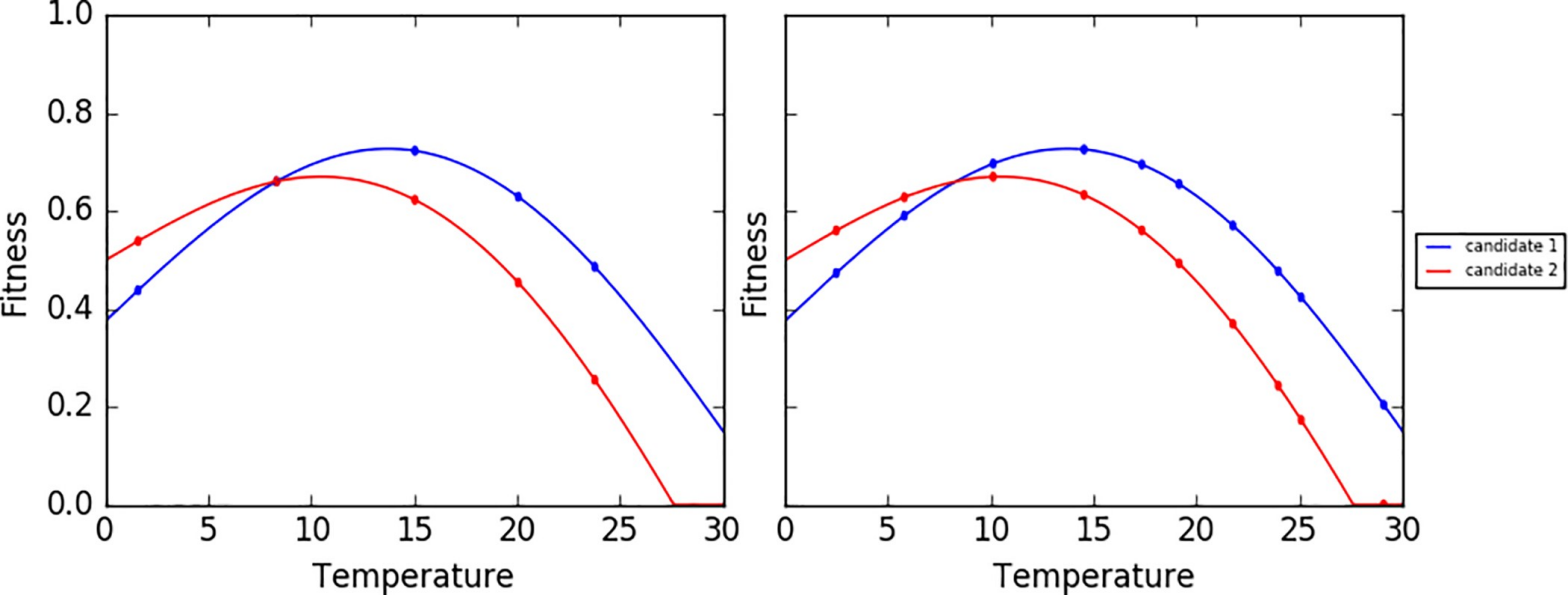
Illustration of the fitness surface of two hypothetical candidate solutions in an environment that varies with respect to a single parameter (e.g. an environment where the temperature varies in the range [0, 30] ^o^C). Assuming that the temperature varies with a uniform distribution, the expected fitness of the two candidate solutions corresponds to the average height of the two curves, i.e. 0.55 and 0.46 for candidate solutions 1 and 2. In general, the higher the number of evaluation episodes, the better the precision of the estimation. The circles in the left and right pictures show the fitness measured during 5 and 10 evaluation episodes, respectively, in which the value of the temperature was set randomly. The offset between the average value of the fitness obtained during the evaluation episodes and the expected fitness amount to 0.036 and 0.021, in the case of the left and right picture respectively.

The number of environmental parameters subject to variations (NP) and the variation range of each parameter are determined by the nature of the problem. The number of evaluation episodes (NEE) is set by the experimenter and can be used to regulate the precision of the fitness estimation: the higher the number of evaluation episodes, the higher the precision of the estimation. The underlying reason is that the expected fitness of a candidate solution in uniformly varying environmental conditions corresponds to the average height of a fitness surface in a NP-dimensional space (as exemplified in Fig 2).

The frequency at which the environmental conditions vary across generations (F) can be set to the maximum value or to a moderate value so that the environmental conditions vary every generation or every few generations, respectively. Indeed, as reported by [34], candidate solutions evolved in environments changing every generation outperform candidate solutions evolved in environmental conditions that remain fixed over generations. Furthermore, candidate solutions evolved in environments that vary every 20/50 generations might outperform solutions evolved in environments that change every generation.

The utilization of the same EVM for all the candidate solutions of a generation guarantees that the individuals of the population are evaluated in the same conditions. In this way it is possible to eliminate the risk of preferring individuals with lower expected fitness that faced easier environmental conditions to individuals with a higher expected fitness that encountered less favorable environmental conditions.

The utilization of a high or moderate frequency of environmental variations across generations allows to achieve good results also when the number of evaluation episodes (NEE) is small thanks to the effect of iterated evaluations of related candidate solutions across generations. Moreover, it permits to reduce the risk to keep selecting candidate solutions that perform well only in the current environmental conditions. Indeed, such solutions tend to be discarded in the succeeding generations as a result of variation of the environmental conditions.

The preferential selection of solutions particularly adapted to the current environmental conditions combined with the variation of environmental conditions across generations leads to a randomly varying selection bias that produce an effect similar to noise on fitness measures. The presence of noise in the fitness measure is not necessarily negative and can even facilitate the evolution of better solutions [40, 41, 42] but produces a reduction of the selective pressure. Consequently, the evolutionary algorithm used should be characterized by a sufficiently strong selective pressure [10].

To identify the best solution of a run, we propose to post-evaluate the best solution of each generation for a sufficiently large number of post-evaluation episodes on the basis of a post-evaluation environmental matrix (PEVM). Unlike EVM, PEVM remains constant across generations. The utilization of post-evaluation permits to identify the candidate solutions that operate well also in environmental conditions that differ from the conditions in which they have been selected. The usage of a single post-evaluation matrix permits to compare candidate solutions on equally difficult conditions. Performing the post-evaluation test at every generation has a cost in term of computation. On the other hand post-evaluating only the individuals of the last generations is insufficient since performance does not necessarily increase monotonically across generations (especially when the environmental conditions vary across generations).

Finally, for the choice of the evolutionary algorithm, we suggest to use gradient estimating evolutionary algorithms, such as CMA-ES and xNES [11, 12, 43], that rely on rather greedy selective processes. These algorithms estimate the local gradient iteratively across generations on the basis of the fitness of the population of candidate solutions and use the gradient to explore promising regions of the search space. Moreover, they optimize the performance of a distribution of parameters rather than the performance of specific vectors of parameters [36]. The utilization of a greedy selective process imposing a strong selective pressure compensates the reduction of the selective pressure caused by the fact that the fitness measured in stochastically varying environmental conditions is influenced by a randomly varying bias. The iterated estimate of the local gradient across generations combined with environmental variations across generations leads to gradient averaged over variable environmental conditions which can favor the generation of solutions robust to environmental variations. The utilization of methods optimizing the performance of a distribution of parameters rather than the performance of specific vectors of parameters fosters the selection of solutions robust to variations of parameters which might display also a certain level of robustness with respect to variations of the environmental conditions.

The novelty of the proposed method lies on the original combination of pre-existing methods and techniques. In particular, in the integrated manner in which the four crucial aspects discussed in the previous section have been addressed.

## 4. Experimental setup

In this section we describe the adaptive problems considered, the evolutionary methods tested, and the methodology used for the evaluation of results.

### 4.1 The extended long double-pole balancing problem

**Fig 3:**
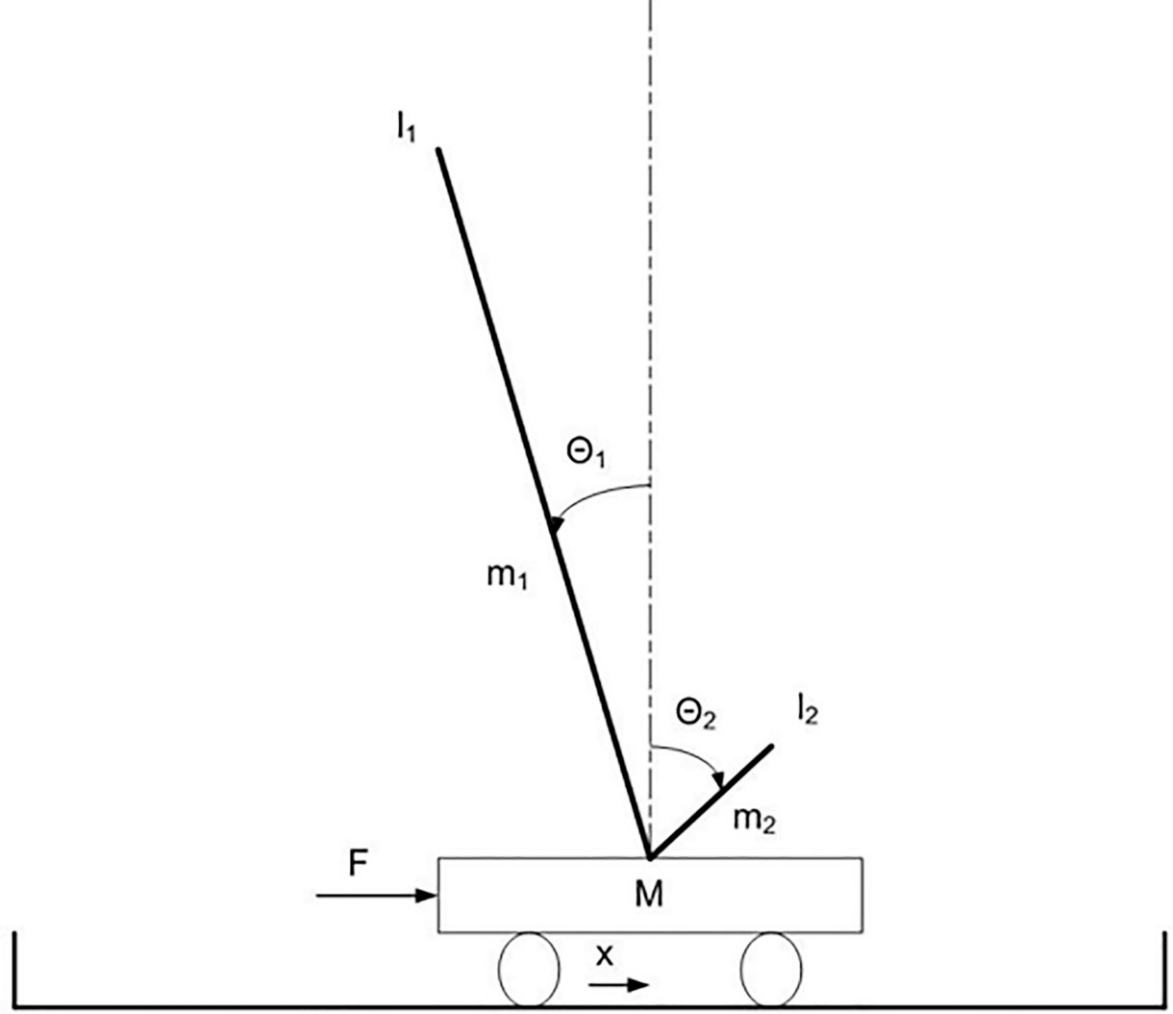
The double-pole balancing problem.

The pole balancing problem, introduced by [44], requires controlling a mobile cart with one or two poles attached through passive hinge joints on the top of the cart for the ability to keep the poles balanced (Fig 3). This problem became a commonly recognized benchmark for continuous control for the following reasons: (i) it involves fundamental aspects of agent’s control (e.g., situatedness, non-linearity, temporal credit assignment [45]), (ii) it is intuitive and easy to understand, and (iii) it requires a low computational cost. Indeed, it has been used to compare the performance of the great majority of the neuroevolutionary methods proposed in the literature (see for example [10, 12, 13, 46, 47, 48, 49]).

The cart has a mass of 1 Kg. The long pole has a mass of 0.5 Kg and a length of 1.0 m. The properties of the second pole are described below. The cart can move along one dimension within a track of 4.8 m. In the non-markovian version of the problem that we consider the cart is provided with three sensors encoding the current position of the cart on the track (x), and the current angle of the two poles (θ_1_ and θ_2_). The motor controls the force applied to the cart along the x-axis. The goal is to maintain the angle of the poles and the position of the cart within a viable range. For a description of the equations used to calculate the dynamics of the system refer to [44].

The controller of the agent is constituted by a fully connected neural network with 4 inputs (3 sensors and 1 bias unit), 10 internal neurons, and 1 motor neuron. In the case of the experiment carried with the NEAT algorithm [13], the networks of the first generation only include the input neurons and the output neuron, but can enlarge across generations as a result of the addition of internal neurons and connections.

The inputs encode the position of the cart (x) and the angular position of the two poles (θ_1_ and θ_2_). The state of the x, θ_1_ and θ_2_ sensors are normalized in the [-0.5,0.5] m, [$‑\frac{5\pi }{13.5},\frac{5\pi }{13.5}$] rad, and [$‑\frac{5\pi }{13.5},\frac{5\pi }{13.5}$] rad ranges, respectively. The activation state of the motor neuron is normalized in the range [-10.0, 10.0] N and is used to set the force applied to the cart. The state of the sensors, the activation of the neural network, the force applied to the cart, and the position and velocity of the cart and of the poles are updated at a frequency of 50 Hz.

The environment in which the agent operates is stable and is constituted by a flat surface of 4.8 m. However, the initial position and velocity of the cart (x, $\dot{x}$), and the initial position and velocity of the poles (θ_1_, ${\dot{\theta }}_{1}$, θ_2_, ${\dot{\theta }}_{2}$), namely the relationship between the agent and the environment, vary.

In the standard version of this problem (see for example [12, 13, 46, 47, 48]) the length/mass of the second pole is set to $\frac{1}{10}$ of that of the first pole and evolving agents are evaluated for a single episode. We thus use the term extended long double pole problem to indicate our version in which the second pole is longer and agents are requested to balance the poles from different initial states. In most of these studies the initial state of the cart does not vary and corresponds to the following values [x = 0.0, $\dot{x}$ = 0.0, θ_1_ = 0.07, ${\dot{\theta }}_{1}$ = 0.0, θ_2_ = 0.0, ${\dot{\theta }}_{2}$ = 0.0]. In the case of [49], instead, the initial state of the single evaluation episode is selected randomly every time within the following intervals: $\left[-2.4<x<2.4,\;\dot{x}=0.0,\;{-0.6<\theta }_{1}<0.6,\;{\dot{\theta }}_{1}=0.0,\;{-0.6<\theta }_{2}<0.6,\;{\dot{\theta }}_{2}=0.0\right]$. Clearly, the utilization of a single evaluation episode does not favor the selection of robust solutions, i.e. agents capable of keeping the poles balanced in environmental conditions that vary freely (within limits) with respect to those parameters. Nevertheless, the evolved agents display a certain level of generalization even in these conditions. This can be explained by considering that, in order to be successful, candidate solutions need to balance the poles also from the states assumed during the course of the episode. However, the states encountered by the evolving agents during the course of their evaluation episode represent only a subset of all possible states. For this reason the utilization of a single evaluation episode does not foster the selection of truly robust solutions, i.e. solutions capable of balancing the poles in the largest possible number of conditions, especially when the state of the agent at the beginning of the evaluation episode is kept constant.

For these reasons, in the experiment reported in this article we used an extended version of the problem in which the agents are required to balance the poles during multiple evaluation episodes in which the cart and the poles assume randomly different initial states chosen within the following intervals [−1.944 < x < 1.944, -1.215 < $\dot{x}<1.215,\;{-0.0472<\theta }_{1}<0.0472,-0.135088<{\dot{\theta }}_{1}<0.135088,\;{-0.10472<\theta }_{2}<0.10472,\;-0.135088<{\dot{\theta }}_{2}$ < 0.135088]. In the majority of previous studies the generalization ability of the evolved networks has been tested by post-evaluating them for 625 trials from initial states in which the x, $\dot{x}$, θ_1_ and ${\dot{\theta }}_{1}$ vary while θ_2_ and ${\dot{\theta }}_{2}$ are always set to 0.0. We decided to post-evaluate the network on 500 trials and vary also the initial state of the second pole in order to increase the variability of the conditions experienced by the agents.

Moreover, to increase the complexity of the problem we set the length/mass of the second pole to $\frac{1}{2}$ of that of the first pole instead of $\frac{1}{10}$ as in the case of the standard version of the problem. Indeed, as pointed out by [44], the longer the second pole, the higher the complexity of the problem.

The aspect that is subjected to variation is the initial agent/environmental relation. In particular, the relative position of the cart, and the orientation and the speed of two poles.

Episodes terminate after 1000 steps or when the angular position of one of the two poles exceeded the range [$‑\frac{\pi }{5},\frac{\pi }{5}$] rad or when the position of the cart exceed the range [-2.4, 2.4] m.

The fitness of the agent corresponds to the fraction of time steps in which the agent maintains the cart and the poles within the allowed position and orientation ranges averaged over multiple evaluation episodes and is calculated on the basis of the following equations:

$$f_{i}=\frac{t}{1000}$$ (1)

$$F=\frac{\sum_{i=1}^{NEE}f_{i}}{NEE}$$ (2)

where *t* is the time step in which the cart or the poles exceeded the allowed range or 1000 in case they are maintained in the range until the end of the trial, *f*_*i*_ is the fitness of a trial, *NEE* is the number of evaluation episodes, and *F* is the total fitness.

The source code for replicating the experiments is available by downloading S1 File.

### 4.2 The car-racing problem

The design of car controllers for The Open Racing Car Simulator (TORCS, [50]) constitutes another commonly used benchmark for continuous control. TORCS (torcs.sourceforge.net) is a state-of-the-art open source car-racing simulator. It combines the features of an advanced commercial simulator with those of a fully customizable research environment. It includes a rather sophisticated physics engine, which takes into account many aspects of the racing car (e.g. collisions, traction, aerodynamics, fuel consumption, etc.) and allows the users to develop their own car controllers (named bots) as separate C++ modules, which can be easily compiled and added to the game. At each control step, a bot can access the current game state, which includes information about the car and the track, and can control the car using the gas/brake pedals, the gear stick, and steering wheel. The game distribution includes many programmed bots, which can be easily customized or extended to build new bots. TORCS users developed several bots, which often compete in international competitions [51].

Machine learning methods applied to this domain usually focus on the problem of identifying an optimal racing line, i.e. a trajectory that the car should follow to minimize the lap-time on a given track with a given car [52]. The low-level control necessary to produce such target trajectory is then performed by a human-designed controller. On the contrary, as in the case of [53], we evolve neural network controllers that learn to drive from scratch and determine the desired state of the actuators of the car on the basis of the state of the game that can be perceived from the car-driver point of view.

The neural network controller is provided with 12 sensory neurons encoding, respectively: (i) the left and right offset of the car with respect to the left and right track edge, (ii) the angular offset between the direction of the car and the orientation of the current track segment, (iii) the current car speed, (iv) the class of the current segment, i.e. 00, 01, and 10 for straight, right curves, and left curves, respectively (v) the left or right angle curvature of the current track segment, (vi) the class of the next segment, (vii) the left or right curvature of the next track segment. All values are normalized in the range [0.0, 1.0]. In the case of the left and right offset of the car, values higher than 0.8 correspond to situations in which the barycenter of the car is outside the track. For a discussion on the importance of perceiving the curvature of the next path segment and a description of how this information can be inferred from the state of distance sensors measuring the distance between the car and the track edge, see [54]. The network controller is also provided with two motor neurons encoding the steering and the acceleration/brake command. The output of the steering neuron is normalized in the range [-1.0, 1.0]. The acceleration/brake neuron is used to accelerate and brake for values higher and lower than 0.5, respectively. The intensity is directly proportional to the state, in the case of acceleration, and inversely proportional to the state, in the case of deceleration.

Gears are controlled automatically by a simple hand-designed gear control that increases/decreases the current gear on the basis the car speed. Initially, the gear is set to 1 and is increased of one unit when the car speed exceeds the following values [67 km/h; 114 km/h; 166 km/h; 212 km/h; 246 km/h] and decreased of one unit when the car speed decrease below the following values [232 km/h; 198 km/h; 152 km/h; 100 km/h; 53 km/h], respectively.

**Fig 4:**
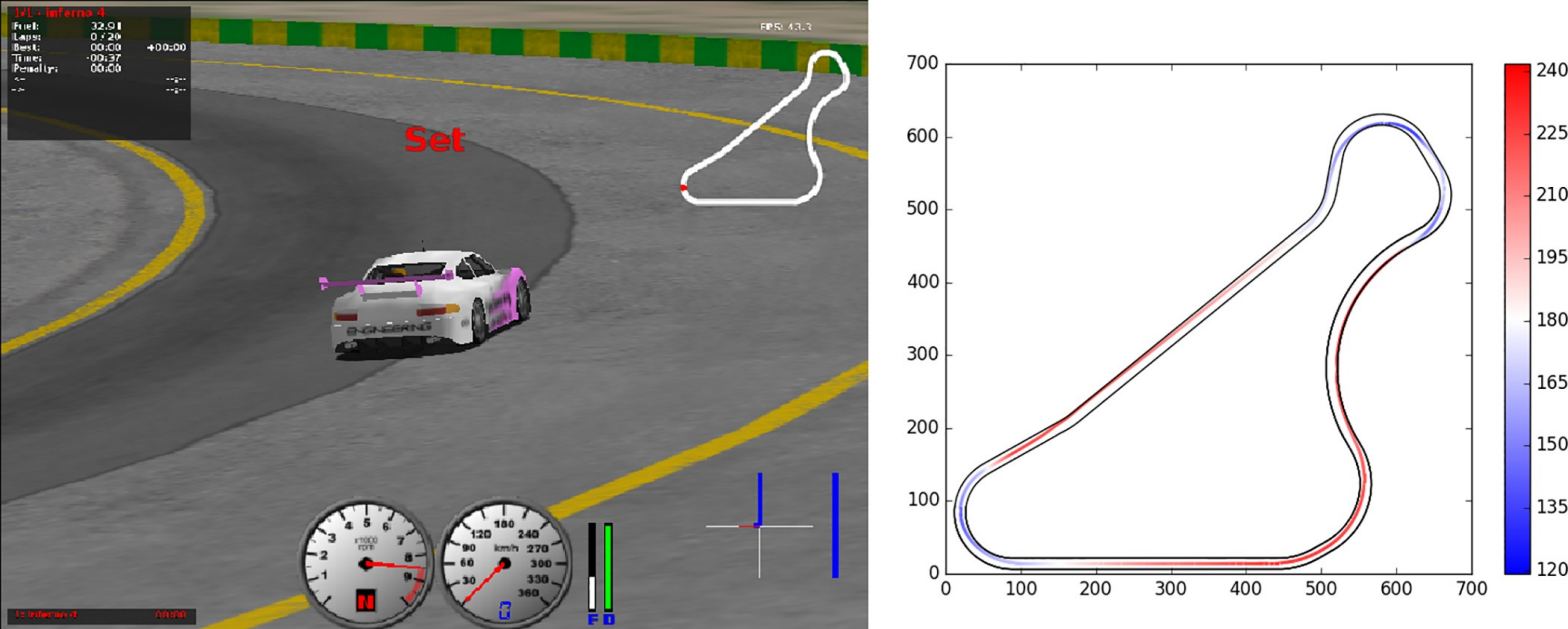
Left: A screenshot of the TORCS graphic render. Right: The CG-Track1 used in the experiments and the trajectory of the best evolved controller (see Section 5.2). The color indicates the speed in km/h during one lap of the circuit.

The experiments have been performed by using the car car2-trb1 and the CG-Track1 circuit (Fig 4). Environmental variations are introduced by setting the position of the start of the race in randomly selected locations of the circuit. Since the speed of the car in each portion of the circuit depends on the distance from the start, evolved controllers should be able to drive effectively independently of the initial speed with which they enter in each portion of the circuit.

Candidate solutions are rewarded on the basis of the distance covered during 5 * 10^4^ simulation steps (corresponding approximately to 200s) and are penalized for running outside the track and for tailspins. More precisely, the fitness is calculated on the basis of the following equation:

$$f_{i}=d*\left(1.0-0.25*\frac{{n_{out}}^{2}}{N}\right)*(1.0-0.25*a)$$ (3)

$$F=\frac{\sum_{i=1}^{NEE}f_{i}}{NEE}$$ (4)

where *d* is the raced distance in m, *n*_*out*_ indicates the number of steps the car is out of the track, *N* is the total number of steps, and *a* is a boolean value indicating whether or not the car produced tailspins, *f*_*i*_ the fitness of an evaluation episode, and NEE the number of evaluation episodes.

The source code for replicating the experiments is available by downloading S2 File.

### 4.3 The swarm robotic foraging problem

Swarm robotics [55] studies a particular class of multi-robot systems, composed of a large number of relatively simple individual robots, and emphasizes aspects like decentralization of control, robustness, flexibility and scalability. Evolutionary Swarm Robotics [56] consists of the utilization of evolutionary methods for the design of this type of systems.

**Fig 5:**
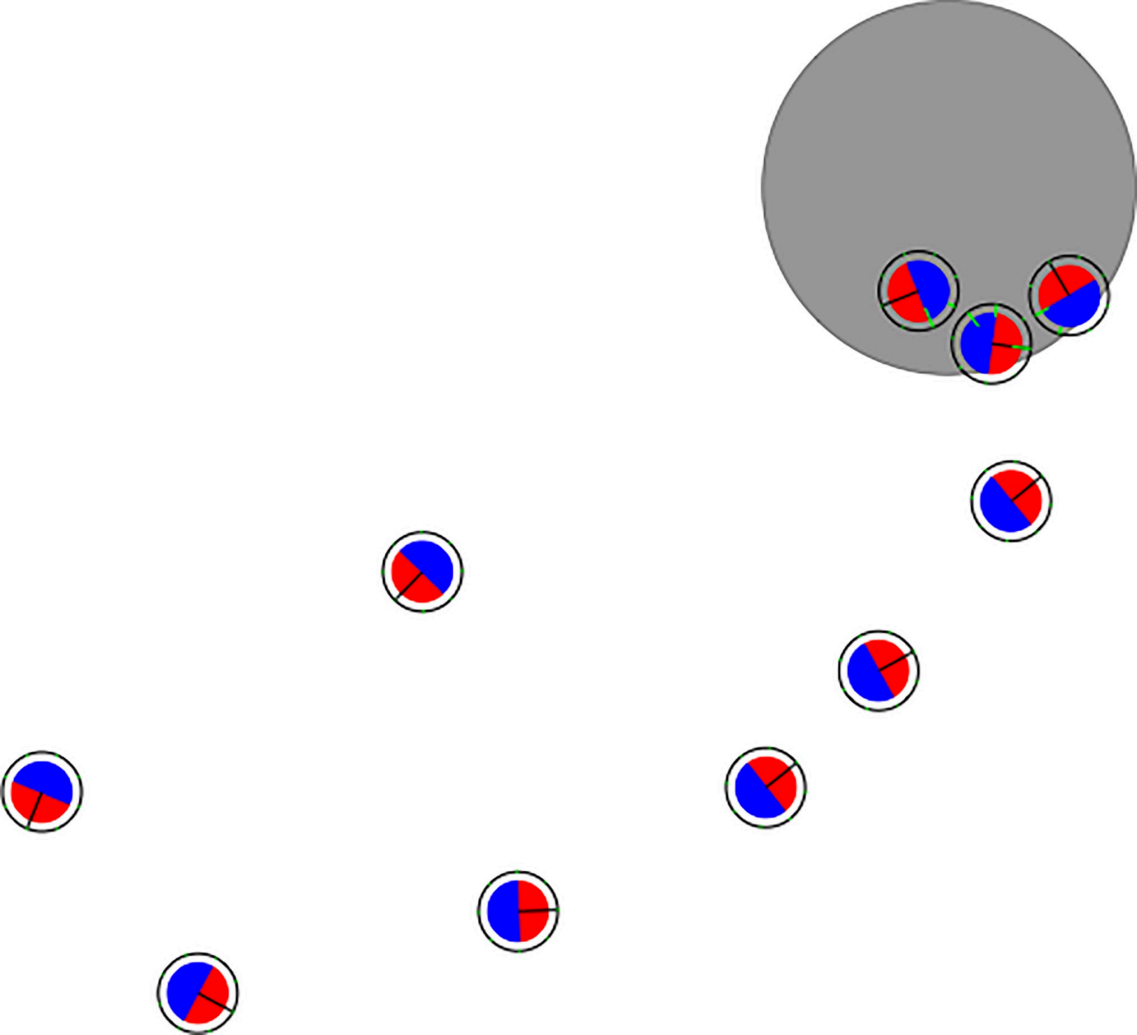
Top view of the environments and of the robots. The black lines and the gray circle represent the walls and the nest, respectively. The smaller circles represent the robots. The red and blue semicircles inside the robot indicate the red and blue LEDs located on the frontal and rear side of the robot.

Here, we consider a collective foraging problem in which a group of 10 simulated marXbot robots [57] should collect food elements and bring them to the nest (Fig 5). The robots are located in a flat square arena of 5 m x 5 m, surrounded by walls, which contains a nest, i.e. a circular area with diameter of 0.8 m painted in gray. The robots, which have a circular shape and a diameter of 0.34 m, are provided with two motors controlling the desired speed of the two corresponding wheels, a ring of LEDs located around the robot body that can be turned on or off and can assume different colors, an omnidirectional camera, 36 infrared sensors located around the robot body that can detect the presence of nearby obstacles, and 8 ground infrared sensors that can be used to detect the color of the ground.

Four hundred elements of invisible food are located inside 400 corresponding 0.5 x 0.5 m non-overlapping portions of the environment. The robots have an energy level that is replenished inside the nest and decreases linearly over time outside the nest. More specifically, the energy level is reset to 1.0 inside the nest and decreased of 0.01 every 100 ms spent outside the nest. To collect food, the energy of the robot should be greater than 0.0. Food elements are automatically collected and released when the robot enters in a portion of the environment containing a food element and in the nest, respectively. The task of the team of robots (swarmbot) consists of collecting and bringing to the nest the higher possible number of food elements within 100 s. This in turn requires that the robots visit the higher possible number of environmental portions by periodically returning to the nest so to release collected food elements and replenish their energy. Fitness corresponds to the number of food elements released in the nest by the robots and is calculated on the basis of the following equations:

$$f_{i}=ne$$ (5)

$$F=\frac{\sum_{i=1}^{NEE}f_{i}}{NEE}$$ (6)

where *ne* indicate the number of food element collected during an evaluation episode, *f*_*i*_ the ftness of an evaluation episode, and NEE the number of evaluation episodes.

The neural network controller of each robot is provided with the following sensory neurons: 8 units encoding the average activation of 8 groups of 4 adjacent infrared sensors, 4 units encoding the fraction of red and blue pixels detected in the frontal-left and frontal-right 45^o^ sectors of the camera, 2 units encoding the state of left and right ground infrared sensors, 1 unit encoding the current simulated energy level, and 1 bias unit. Moreover, the controller is provided with the following motor neurons: 2 units encoding the desired speed of the robot’s left and right wheels, and 2 units binarily encoding whether or not the 16 frontal LEDs are turned on in red and whether or not the 16 rear LEDs are turned on in blue.

The swarmbot is formed by identical individuals. Each candidate solution encodes the characteristics of a single neural controller that is replicated 10 times and embedded into the 10 corresponding robots.

At the beginning of each evaluation episode: (i) the nest is placed in a randomly chosen location inside the arena at a distance of at least 1 m from the walls, (ii) the 10 robots are located in a randomly different positions and orientations in an area of 1 x 1 m centered on the location of the nest, (iii) the environment is replenished with 400 food elements located in 0.5 x 0.5 m non overlapping portions of the arena. Therefore, the evolving controllers should allow the 10 robots to forage effectively irrespectively of the initial condition. Since the positions assumed by the robots during the course of the episode are influenced by their initial positions and orientations, evolved robots should be able to forage effectively in a wide range of different conditions.

In the case the three considered problem domains, performance is evaluated on the basis of the best fitness of the run and on fitness distribution among multiple runs. We intentionally avoided to evaluate performance by measuring how often or how quickly near-optimum solutions are found. For a critical analysis of the latter approach, see [58, 59, 60].

The source code for replicating the experiments is available by downloading S3 File.

### 4.4 The evolutionary algorithms

To verify the possibility of evolving robust solutions for these problems and checking the relative efficacy of alternative methods in this context we carried out the experiments with five different algorithms. Four methods are used to evolve the connection weights of neural networks with fixed topologies. Instead, the fifth algorithm permits to evolve both the connection weights and the topology of the neural network.

The first used method consists of a standard (μ+μ) evolutionary strategy [9, 35] that we named **stochastic steady state** (**SSS**, [10]). We choose this method since it outperformed several alternative methods on two variants of the standard double-pole balancing problem in which the candidate solutions were evaluated for the ability to balance the poles from variable initial states and in which the update of sensory states was delayed of 20 ms [10]. It operates on the basis of populations formed by μ parents. During each generation each parent generates one offspring, the parent and the offspring are evaluated, and the best μ individuals are selected as new parents. Variations are introduced by mutating each policy parameter with a given probability and are performed by either perturbing the value with a Gaussian distribution or replacing the value with a new random value selected with a uniform distribution. The term stochastic refers to the fact that the method includes the possibility to perturb the fitness with the addition of a randomly selected value. The parameters that should be set manually are the population size, the mutation probability, the range of the connection weights, and the range of the noise added to the fitness (when used). For a detailed description see [10].

The second considered method is the **covariance matrix adaptation evolution strategy** (**CMA-ES**, [11]), a state-of-the-art continuous optimization method. It is a form of evolutionary strategy (ES) characterized by deterministic truncation selection, unbiased variation operators and self-adaptation, i.e. change of the behavior of variation operators during the course of the evolutionary process. Instead of an explicit population, CMA-ES maintains a multivariate Gaussian probability distribution N(m, σ^2^C) where m and C are its mean and covariance matrix while σ is a step-size. In each iteration, the distribution is used to sample λ new candidate solutions, which are then evaluated. Afterwards, the three parameters of the distribution are updated on the basis of the observed ranking of the candidate solutions. The new mean m is calculated as a weighted recombination of the best μ candidate solutions. The update procedures of covariance matrix C and step-size σ are more complex. For a detailed description the reader is referred to the original work of [11]. The population size can be set automatically on the basis of the number of parameters [11]. Consequently, this method does not require to manually set any parameter.

The third considered method is the **exponential natural evolutionary strategy** (**xNES**) [43], a prominent member of the family of the natural evolutionary strategies (NES) [12]. xNES uses the Gaussian mutation to generate new search points and adjusts the parameters of the mutation at each generation in order to improve the expected fitness under the mutation distribution. For the adjustment, the xNES makes use of the natural gradient [49,61] of the expected fitness with respect to the parameters of the mutation distribution, which is referred to as a natural evolution gradient. CMA-ES and xNES are closely related and operate in a similar manner. Indeed, as demonstrated by [50,62], natural evolution strategies can be viewed as a variant of covariance matrix adaptation evolution strategies. The step-size can be set to 1.0 and the population size can be set automatically on the basis of the number of parameters. Consequently, this method does not require to manually set any parameter. For a detailed description of the method, see [43]. We choose this method since it is competitive with respect to CMA-ES on black-box optimization benchmarks [43] and since it resulted one of the best in the comparative analysis reported in [7,10].

The fourth examined method is the **separable natural evolutionary strategy** (**sNES**, [43]), another instance of natural evolutionary strategies. The utilization of a separable and linear search distribution makes this method more suitable for problems involving high-dimensional search spaces [43]. For a detailed description of the method see [43].

Finally, the fifth analyzed method is the **neuroevolution of augmenting topologies** (**NEAT**) [13], a method devised specifically for the evolution of neural networks. We choose this method because it is widely used for the evolution of neural networks and allows to optimize not only the connection weights, but also the topology of the network. The initial population is composed of a vector of μ genotypes of equal length encoding minimal networks in which input neurons are directly connected to output neurons. Each gene contains four numbers representing the ID number of the neurons sending and receiving the connection, the weight of the connection, and the history marker constituted by a progressive innovation number. The length of the genotype and the size of the neural network grow across generations. This occurs through the usage of an add-node genetic operator, which replaces a gene encoding a connection with two genes that encode a new connection to and from a new internal neuron, and an add-connection operator that adds a new gene encoding the connection between two pre-existing neurons. The method also relies on innovation identification numbers associated to genes, which permit to cross over networks with varying topologies, and on the utilization of speciation and fitness sharing [63] to preserve innovations. For a detailed description of the method, see [13]. NEAT requires setting many parameters. In the experiment reported below we used the parameter values recommended by the authors [13]. The authors later proposed new extended methods such as HyperNEAT [64, 65] that are targeted to the evolution of large neural networks.

In the case of CMA-ES, xNES and sNES the values of the connection weights are unbounded and initialized in the range [-1.0, 1.0]. In the case of the SSS and NEAT the connection weights are bounded and initialized in the range [-8.0, 8.0]. This corresponds to the value suggested by [13] for NEAT.

### 4.5 Evaluation of results

We will use the term fitness to indicate the average score obtained during the evaluation episodes and the term performance to indicate the average score obtained during the post-evaluation episodes (see Section 3). Performance indicates the ability of the agents to operate in varying environmental conditions, i.e. the ability of the agents to generalize in different environmental conditions.

The evolutionary process is continued until a total maximum number of evaluations have been performed. This number include both evaluation of candidate solutions and post-evaluations of the best individual of each generation. Since the evaluation and the post-evaluation of candidate solutions constitutes the major computational cost, this design choice allows us to compare experiments in which the number of evaluation episodes or the population size differs by maintaining the computational cost approximately constant.

Statistical differences among performance are analyzed by using the Mann-Whitney U test and Bonferroni correction in the case of multiple comparisons.

## 5. Results

### 5.1 The extended long double-pole balancing problem

Table 1 displays the average performance obtained in the extended long double-pole balancing problem with the five evolutionary methods considered. The total number of evaluations was set to 32 * 10^6^. NEE and NVE were set to 20 and 500. Each experiment has been replicated six times by varying the frequency at which the environmental conditions varied across generations. The optimal performance for this problem is unknown but it is lower than 1.0 since balancing the poles from certain initial states is likely impossible.

**Table 1:** Performance of the best solutions post-evaluated for 500 validation episodes for experiments in which the environmental conditions vary with different frequency across generations. F = 1 / G, where G is the number of generations after which the environmental conditions vary. Experiments conducted with NEE = 20. Each number indicate the average result of 40 replications. Numbers in brackets indicate the standard deviations.

	F = 0.0	F = 0.01	F = 0.02	F = 0.04	F = 0.1	F = 1.0
**SSS**	0.043 [0.005]	0.269 [0.084]	0.313 [0.078]	0.332 [0.090]	**0.335** [0.109]	0.151 [0.088]
**CMA-ES**	0.364 [0.079]	0.510 [0.084]	0.549 [0.060]	**0.571** [0.078]	0.570 [0.072]	0.455 [0.084]
**xNES**	0.521 [0.038]	0.536 [0.044]	0.554 [0.037]	0.555 [0.036]	0.552 [0.036]	**0.567** [0.034]
**sNES**	0.325 [0.063]	0.376 [0.079]	0.388 [0.077]	0.384 [0.087]	0.442 [0.077]	**0.483** [0.079]
**NEAT**	0.174 [0.082]	0.191 [0.097]	**0.194** [0.090]	0.174 [0.090]	0.165 [0.079]	0.166 [0.070]

All methods, with the exception of NEAT, achieve better performance when the environment varies across generations [0.01 > = F < = 1.0] than when the environment remains stable [F = 0.0] (Table 1, p-value < 0.05). In the case of the CMA-ES and SSS, the best performance is achieved with intermediate frequencies: [0.02–0.1] (p-value < 0.05 for comparisons of the conditions inside the interval with the conditions outside, and p-value > 0.05 for comparisons among conditions inside the interval). These results are in line with the analysis carried on the SSS reported in [34]. Instead, in the case of xNES and sNES the best performance is achieved with high frequencies. Indeed, in the case of xNES the F = 1.0 condition outperforms the F = 0.0 and F = 0.01 conditions (p-value < 0.05) and does not differ statistically from the F = 0.02, F = 0.04, and F = 0.1 conditions (p-value > 0.05). In the case of sNES, the F = 1.0 condition outperforms all other conditions (p-value < 0.05).

Table 2 displays the performance obtained by varying systematically the number of evaluation episodes from 1 to 1000. Performance is maximized in experiments carried with the following NEE: [20, 50] for SSS, [20, 50, 100, 200] for CMA-ES, [50, 100, 200] for xNES, [20, 50, 100, 500, and 1000] for sNES, and [10, 20, 50, 100] for NEAT. The performances in the conditions indicated in the above intervals differ statistically from the other conditions (p-value < 0.05) and do not differ statistically among themselves (p-value > 0.05). These results indicate that the utilization of a relatively small number of evaluation episodes (i.e. 50) is sufficient to maximize performance irrespectively of the method used. Due to the tradeoff between the number of generations and number of evaluation episodes, the optimal NEE might increase and decrease, respectively, in experiments in which the total number of evaluations episode is larger or smaller. However, the fact that in these experiments the total number of evaluations episodes used is rather large suggests that the optimal number of environmental conditions experienced by each candidate solution during its evaluation is relatively small.

**Table 2:** Performance of the best solutions post-evaluated for 500 validation episodes for experiments in which NEE is varied from 1 to 1000. F is set to 1.0 (i.e. the ENV matrix is re-generated every generation). Each number indicate the average result of 40 replications. Numbers in brackets indicate the standard deviations.

	NEE = 1	NEE = 10	NEE = 20	NEE = 50	NEE = 100	NEE = 200	NEE = 500	NEE = 1000
**SSS**	0.099 [0.015]	**0.213** [0.098]	0.151 [0.088]	0.123 [0.073]	0.091 [0.055]	0.085 [0.049]	0.082 [0.042]	0.088 [0.039]
**CMA-ES**	0.125 [0.060]	0.413 [0.104]	0.455 [0.084]	0.477 [0.069]	0.483 [0.063]	**0.504** [0.062]	0.441 [0.074]	0.412 [0.073]
**xNES**	0.087 [0.062]	0.508 [0.028]	0.567 [0.034]	0.605 [0.035]	**0.607** [0.035]	0.600 [0.038]	0.558 [0.040]	0.517 [0.031]
**sNES**	0.139 [0.097]	0.421 [0.074]	0.483 [0.079]	0.500 [0.052]	**0.507** [0.069]	0.484 [0.072]	0.475 [0.066]	0.465 [0.068]
**NEAT**	0.068 [0.016]	0.153 [0.068]	**0.166** [0.070]	0.150 [0.060]	0.138 [0.058]	0.120 [0.040]	0.083 [0.021]	0.064 [0.008]

**Fig 6:**
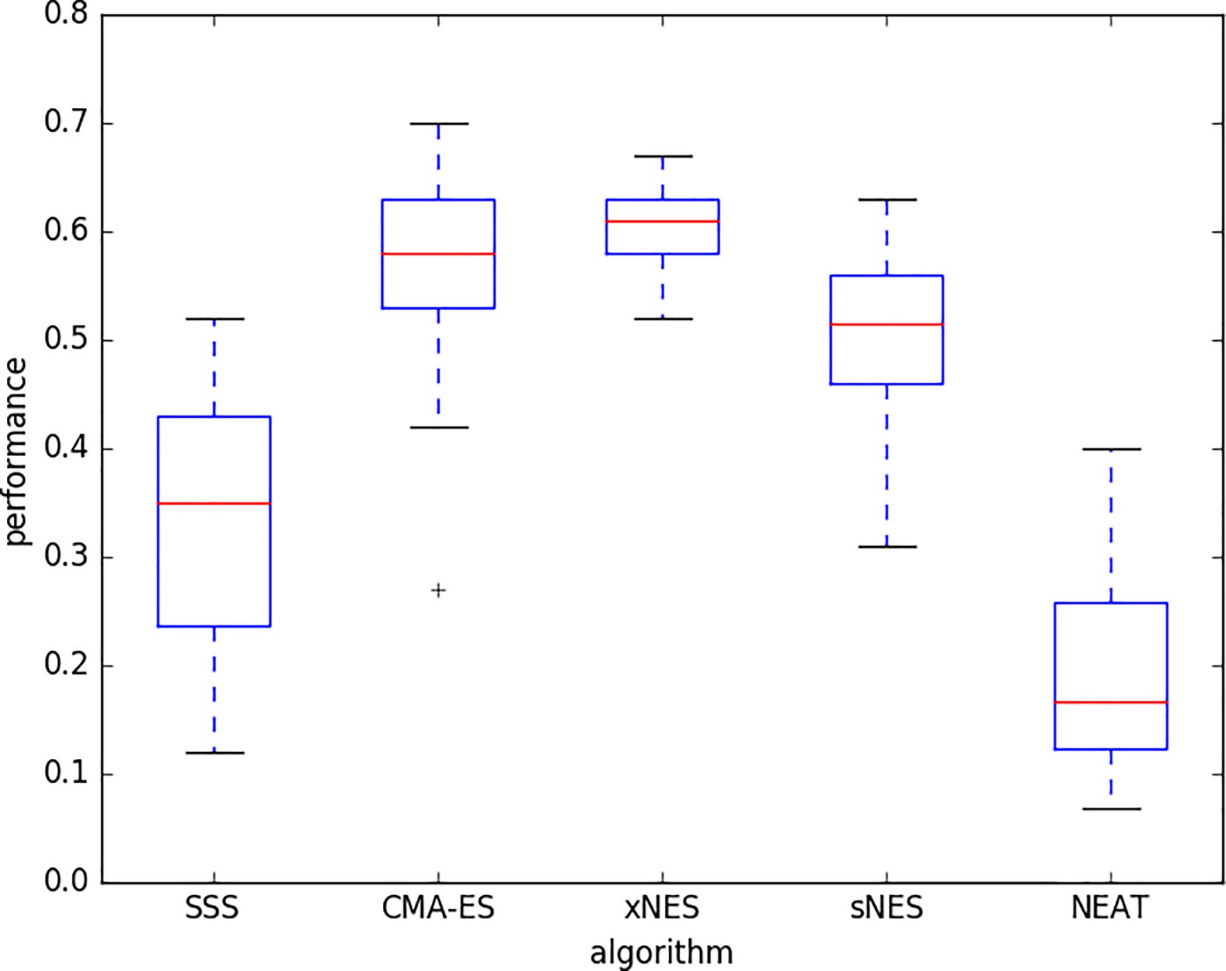
Performance of the best candidate solutions post-evaluated for 500 trials. Experiments performed with the best combination of parameters for each algorithm: SSS (F = 0.1, NEE = 20); CMA-ES (F = 0.04, NEE = 20); xNES (F = 1.0; NEE = 100); sNES (F = 1.0, NEE = 100); NEAT (F = 0.02, NEE = 20). Boxes represent the inter-quartile range of the data and horizontal lines inside the boxes mark the median values. The whiskers extend to the most extreme data points within 1.5 times the inter-quartile range from the box. “+” indicate the outliers. Data obtained by running 40 replications.

Fig 6 displays the performance obtained with the five methods with the best combination of parameters. The best performance is achieved by CMA-ES and xNES that outperform the other three methods (p-value < 0.05) and do not statistically differ among themselves (p-value > 0.05). The second in the ranking is the sNES method that is outperformed by CMA-ES and xNES (p-value < 0.05) and outperforms SSS and NEAT (p-value < 0.05). The third in the ranking are the SSS and NEAT methods that are outperformed by CMA-ES, xNES, and sNES (p-value < 0.05) and do not differ among themselves (p-value > 0.05).

**Fig 7:**
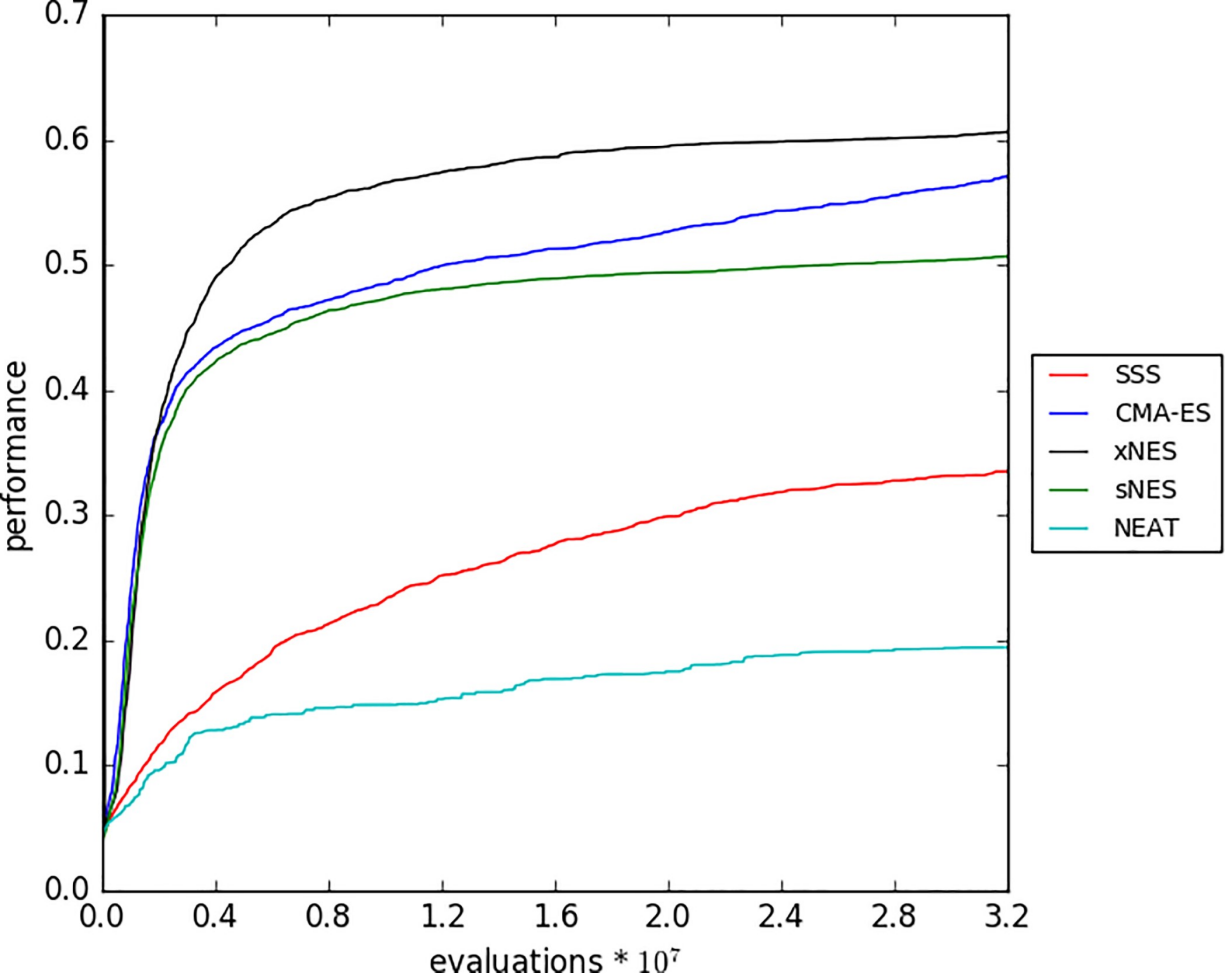
Performance of the best candidate solution so-far across generations for different methods. The X-axis indicate evaluations. Data obtained in the experiments with the best combination of parameters (see caption of Fig 6). Each curve indicates the average results of 40 replications.

The analysis of the best-to-date performance across generations shows how xNES, CMA-ES, and sNES outperform the other two methods from the very beginning for the entire course of the evolutionary process (Fig 7).

**Fig 8:**
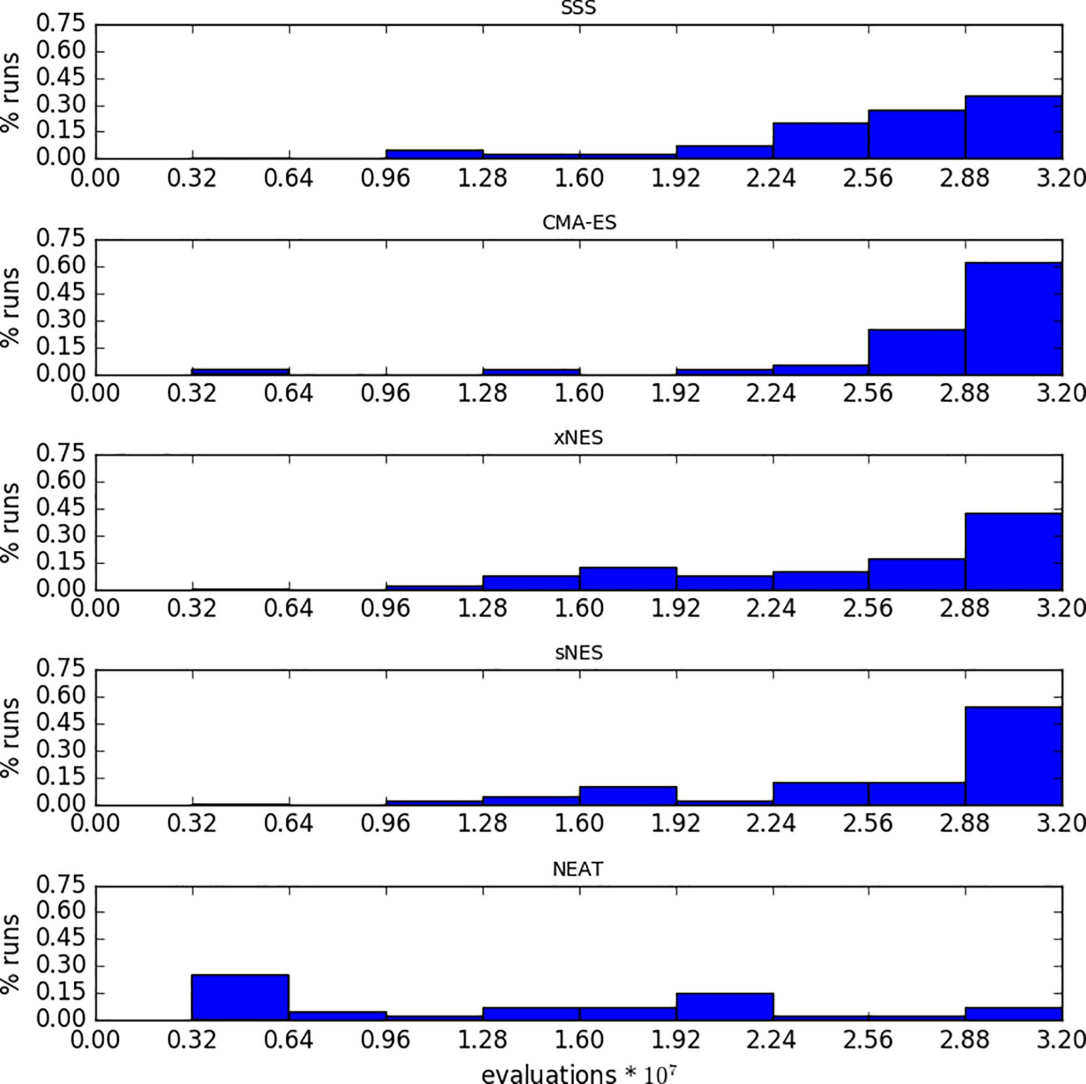
Fraction of times in which the best solution was generated during one of ten consecutive phases of the evolutionary process. Each phase corresponds to 32 * 10^5^ evaluations for a total of 32 * 10^6^ evaluations. Data computed on the experiments performed with the SSS, CMAES, xNES, sNES, and NEAT methods with the best combination of parameters (see Fig 6). Each histogram shows the fraction of runs, out of 40, in which the best solution was found during the corresponding phase.

Fig 8 shows the percentage of times the best solution of a run is generated in one of the 10 consecutive phase of the evolutionary process. As expected, in the majority of the cases the best solution of a run is generated during the final phases of the evolutionary process (with the exception of NEAT). However, in a non-negligible fraction of the runs, the best solution is generated during intermediate and even initial phases of the evolutionary process. This confirms that post-evaluating the best candidate solution of each generation is necessary to identify the truly best solution of a run.

### 5.2 The car-racing problem

**Fig 9:**
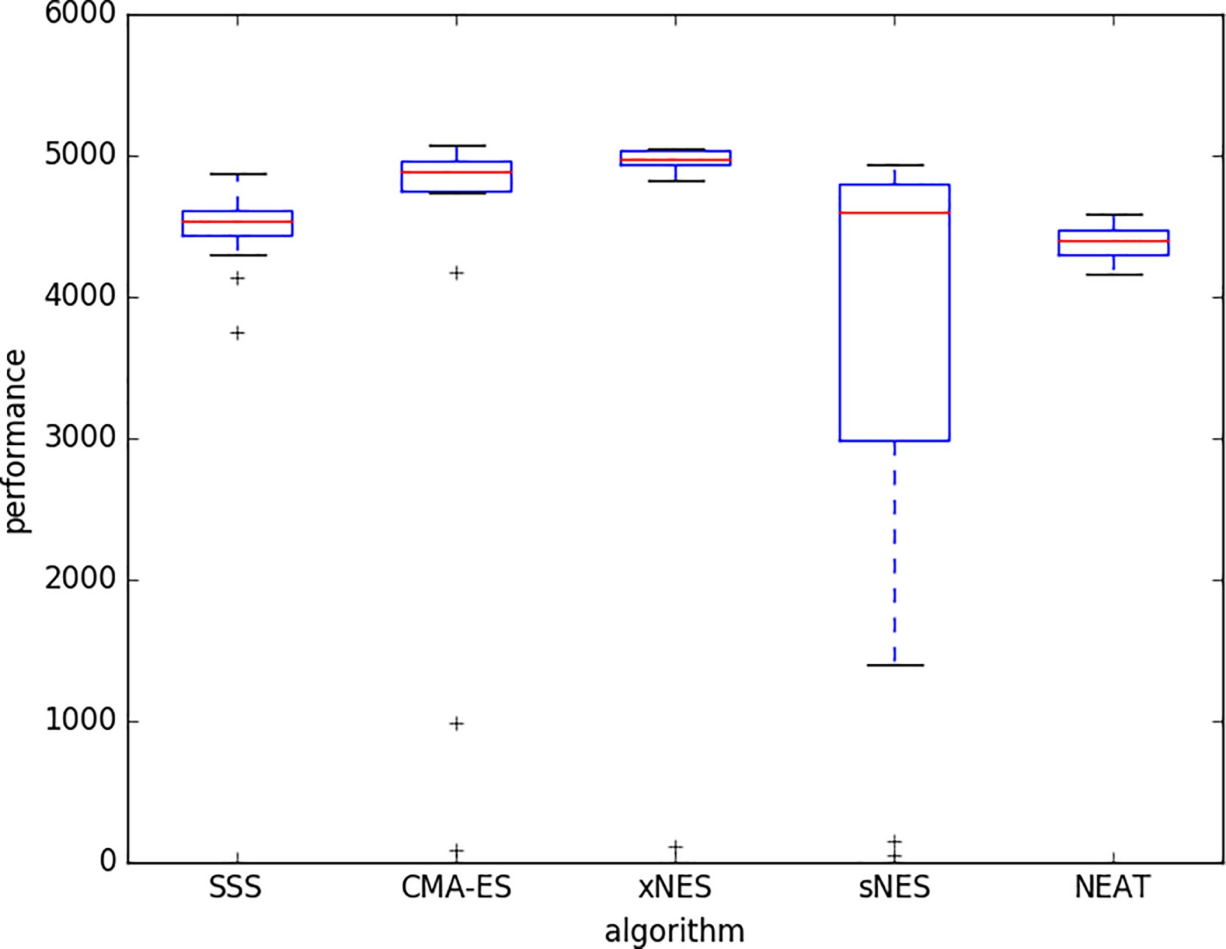
Performance of the best candidate solutions post-evaluated for 25 trials. Each experiment has been replicated 20 times. Boxes represent the inter-quartile range of the data and horizontal lines inside the boxes mark the median values. The whiskers extend to the most extreme data points within 1.5 times the inter-quartile range from the box. “+” indicate the outliers.

Fig 9 displays the performance obtained in the car racing problem with the five evolutionary methods considered. The total number of evaluations was set to 1 * 10^6^. NEE and NVE were set to 3 and 25, respectively. The frequency of environmental variation (F) was set to 0.05, i.e. to a value that produced good performance with the different methods, on the average, in the case of the extended long double-pole problem. Each experiment has been replicated 20 times.

Also in this case the best performance is achieved with the xNES and CMA-ES methods that outperform the sNES, SSS, and NEAT methods (p-value < 0.05) and do not differ among themselves (p-value > 0.05). The performance of the sNES, SSS and NEAT methods do not statistically differ among themselves (p-value > 0.05).

The optimal performance for this problem is unknown. Remarkably, the best controllers obtained with the CMA-ES, sNES, xNES and SSS methods outperform the best human-designed controller named “Inferno” included in the TORCS repository (Table 3).

**Table 3:** Performance of the best controllers evolved with the 5 methods and of the best human-designed driver available in TORCS v1.3.7 called “Inferno” (i.e. the human programmed controller that achieves the best performance on the CG1-track circuit, see http://torcs.sourceforge.net/index.php?name=News&file=article&sid=100). All controllers, including Inferno, have been tested and evaluated in the same conditions.

CMA-ES	xNES	sNES	SSS	Inferno	NEAT
**5078.018**	5047.128	4918.661	4860.633	4658.589	4581.371

Inferno has access to the full description of the circuit and operates by initially computing an optimal racing line. The program then determines the state of the steering, the acceleration and brake actuators of the car at every time-step on the basis of the offset between the current state of the car and the future desired state of the car along the optimal racing line and on the basis of the current speed of the car. How the state of the actuators of the car are controlled is also regulated on the basis of phase of the race (starting versus normal phase) and the risk of penalties (dangerous versus non-dangerous context). Gears are controlled on the basis of the same routine used by evolving controllers and described in Section 4.2.

Also in the case of this problem, the utilization of a limited number of evaluation episodes is sufficient to evolve controllers able to generalize with respect to the initial position of the car in the circuit.

### 5.3 The swarm robotic foraging problem

**Fig 10:**
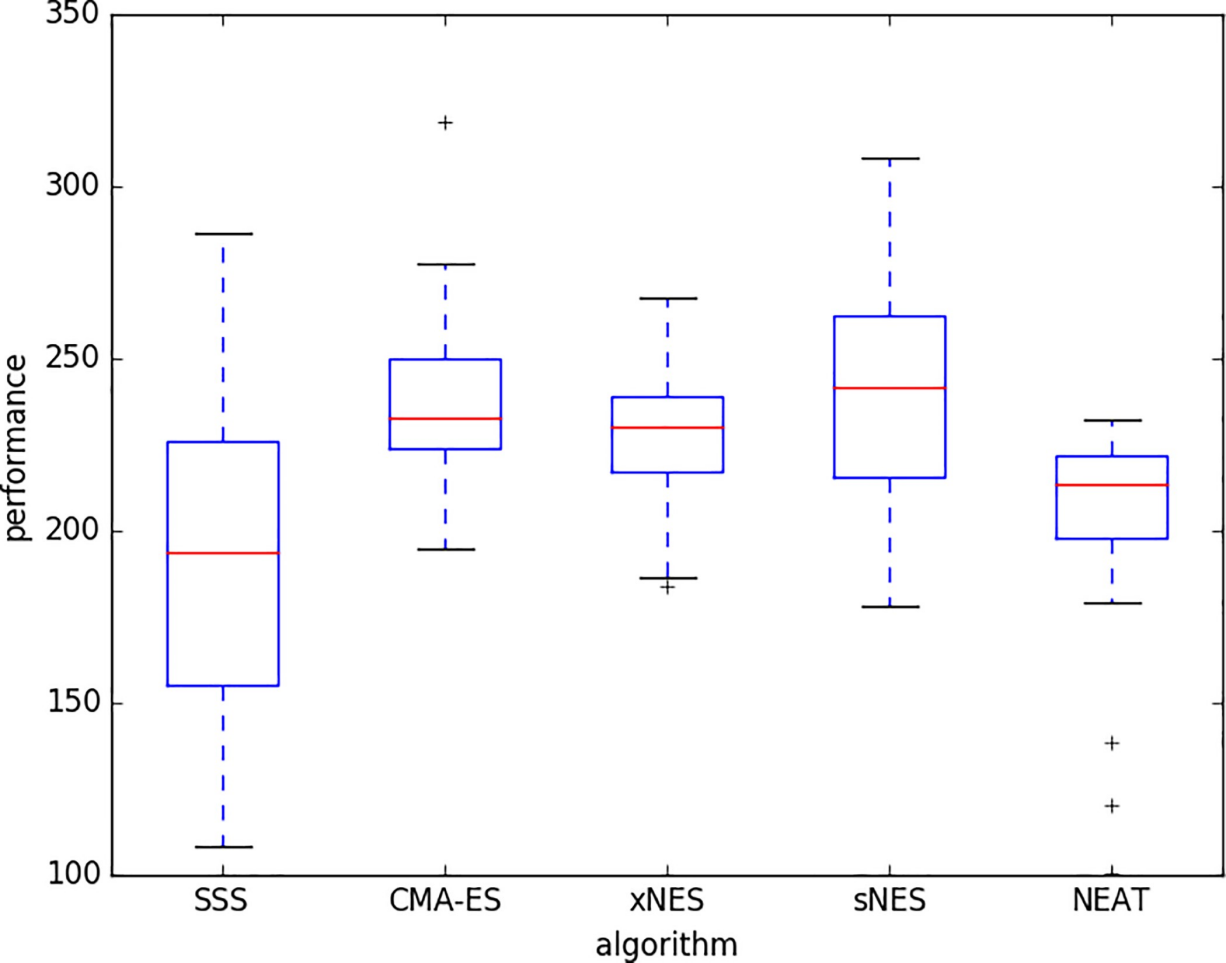
Performance of the best solution post-evaluated for 20 episodes. Boxes represent the inter-quartile range of the data and horizontal lines inside the boxes mark the median values. The whiskers extend to the most extreme data points within 1.5 times the inter-quartile range from the box. “+” indicate the outliers. Data have been obtained by running 30 replications.

Fig 10 displays the performance obtained on the swarm robotic foraging problem with the five evolutionary methods considered. The total number of evaluations was set to 1.5 * 10^6^. NEE and NVE were set to 6 and 20, respectively. The frequency of environmental variation (F) was set to 0.05, i.e. to a value that produced good performance with the different methods, on the average, in the case of the extended long double-pole problem.

In this problem the best performance is achieved by the CMA-ES, xNES, and sNES methods that outperform the SSS and NEAT methods (p-value < 0.05) and do not statistically differ among themselves (p-value > 0.05). The SSS and NEAT methods do not statistically differ among themselves (p-value > 0.05).

The analysis of the behavior displayed by the best evolved robots indicates that evolution manages to discover rather effective and elaborated solutions. The most common and effective solution type involves the formation of a dynamic chain in which the robots arrange themselves in an ellipsoid shape (Fig 5) and move at high speed approximately toward the position of the next robot so to maintain the ellipsoid formation. One side of the ellipsoid chain is located over the nest so the robots can periodically release the collected food items in the nest. The location of the other side of the chain tends to move slowly over time, thus enabling the swarmbot to collect food elements located in different areas of the environment (see S4 File). A typical strategy used by the robots to ensure that one side of the ellipsoid remains anchored on the nest consists of slowing down when they enter in the nest and exiting from the nest only when they detect another robot nearby on their rear side (see S5 File). A common strategy used to ensure that the second side of the ellipsoid varies its position consists of re-entering in the nest after a complete lap of the ellipsoid on the left or on the right of the previous time. Indeed, this strategy guarantees that the second side of the ellipsoid slowly moves clockwise or counter-clockwise over time (see S4 File). Robots relying on this type of strategy turn their rear blue LEDs on and use the perception of blue to form and move along the ellipse of the dynamic chain.

A second class of strategies involves the subdivision of the swarm in two sub-groups of robots: a larger group collecting food and a smaller group (eventually composed of a single robot) marking the position of the nest (see S6 File). The larger group of food collectors is constituted by individuals that move independently in different directions collecting food. The smaller group of nest-marker acts as a beacon and allows the former robots to both infer the position of the nest by distance and to navigate back to the nest in order to release the collected foods and replenish the energy level. The role of the individual robots might change dynamically since food-collectors returning to the nest can become nest-markers and nest-markers might exit from the nest and become food-collectors. The transition between the two roles is regulated so to guarantee that the number of robots playing the role of nest-marker is small.

The behavior of evolved robots is rather robust. They manage to: (i) form the dynamic chains or to subdivide in food-collectors and nest-markers during all trials, (ii) avoid colliding with obstacles and other moving robots during the large majority of the evaluation episodes, and (iii) collect and bring to the nest a significant number of food elements in all trials, irrespectively of both their initial positions and orientations, and the relative positions and orientations of the other individuals. In the case of the robots displaying the dynamic chain strategy, occasionally part of the robots might exit from the chain and form a sub-chain that usually later re-merges with the main chain.

## 6. Conclusions

We investigated the possibility of evolving neural networks that are robust with respect to variations of the environmental conditions and, consequently, operate effectively in new conditions immediately, without the need to adapt to variations. After reviewing related research and discussing the open research issues, we proposed a suitable method that specifies how the fitness of candidate solutions can be estimated, how the environmental conditions should vary to facilitate the evolution of effective solutions, and how the best solution of a run can be identified. Moreover, we analyzed the characteristics that make evolutionary algorithms suitable for the evolution of solutions robust to environmental variations.

The method is characterized by the following aspects: (i) candidate solutions are evaluated multiple times in a limited number of randomly different environmental conditions, (ii) the candidate solutions of a given generation are evaluated in the same environmental conditions, (iii) the environmental conditions change across generations with a given frequency, (iv) the best solution of a run is identified by post-evaluating the best candidate solution of each generation on an independent set of randomly different environmental conditions. The identification of algorithms suitable to evolve robust solutions has been made by comparing the efficacy of five different state-of-the-art evolutionary algorithms with the method described above.

The results of the comparison indicate that algorithms optimizing a distribution of parameters (i.e. CMA-ES, xNES, and sNES) outperform algorithms optimizing a particular vector of parameters (i.e. SSS and NEAT). The advantage of the former methods can be due to their ability to estimate the gradient of the fitness and select solutions robust with respect to variations of their parameters [36]. This can be explained by considering that robustness to parameter variation correlates positively with robustness to variation of the environmental conditions [37]. Future research should investigate the relative importance of gradient estimation and robustness to variations of parameters for the synthesis of solutions robust to environmental variations.

More specifically, the CMA-ES and xNES algorithms outperform the SSS and NEAT methods in all considered problem domains. The sNES algorithm works as well as the CMA-ES and xNES methods in one problem, but produces lower performance in the other two problems.

The results obtained on three qualitatively different problems involving a relatively large number of parameters to be optimized show how the method proposed is effective and computational tractable, i.e. it permits to evolve solutions operating effectively in a wide range of environmental conditions and relying on a limited number of evaluation episodes. It allows to: (i) achieve better performance on the extended long double-pole balancing problem with respect to previous research, (ii) evolve controllers outperforming the best available human-designed controller in the case of the car-racing problem, and (iii) synthesize remarkable effective solutions in the case of the swarm robotic foraging problem (see Section 5.1, 5.2 and 5.3). The applicability of the method to problems significantly more complex than those considered in this paper should be verified in future studies.

Promising future research directions include the development of variations of CMA-ES and xNES algorithms specifically tailored for neuro-evolution. In particular, we believe that the possibility to increase the size of the neural network across generations through a mechanism analogous to that used by NEAT [13] and by SUNA [66] and weight decay mechanisms preventing an excessive growth of parameters might lead to even higher performance. Another interesting aspect deserving further investigation in future research is constituted by behavioral plasticity defined as the ability of agents to display multiple behavioral responses which might differ in a continuous or discontinuous way, in a condition-sensitive manner [5, 67]. Indeed, the ability to display an articulated behavioral repertoire combined with the ability to select immediately the behavior appropriate to the current perceived circumstances can support the synthesis of highly robust solutions. The evolution of behavioral plastic agents can be promoted through the evolution of modular controllers [68–69] and the inclusion of mechanisms facilitating the perception of behavioural affordances, i.e. the perception of states eliciting the execution of specific behavioral responses [5].

## Supporting information

S1 File Source code of the double-pole balancing problem. (ZIP)

S2 File Source code of the car-racing problem. (ZIP)

S3 File Source code of the swarmbot foraging problem. (ZIP)

S4 File An example of evolved swarmbot displaying a dynamical chain strategy. (ZIP)

S5 File A second example of evolved swarmbot displaying a dynamical chain strategy. (ZIP)

S6 File An example of swarmbot that subdivides itself in two subgroups playing the role of nest markers and food collectors. (ZIP)
